# Trichinosis

**Published:** 1886-03

**Authors:** 


### TRICHINOSIS.

There are eight persons in the hospitals of this city suffering from this disease. A number of others also have been afflicted with it, all of whom received the infection while attending a birth-day party at which a boiled ham was a portion of the feast. The disease is caused by eating pork in which trichinæ exist and which has not been sufficiently cooked. The meat must be subjected to a temperature of at least 150 degrees all through to kill the parasite. The trichina is a little white worm or "wiggler," so small that it canno be seen with the naked eye, and is supposed to originate in the rat, though some medical authorities hold that it is also natural to the hog. However that may be, hogs get the disease and their flesh becomes filled with these parasites. So tenacious of life are the trichinæ that they live and flourish so long as the pork is preserved from decomposition. A ham, for instance, which has been smoked too hurriedly and not subjected to a sufficient heat, will preserve them for months. Other animals may be impregnated with the disease by taking into the stomach meat in which the trichinæ exist, and it is believed that it is in this way that it is conveyed from the rat to the hog.

The development of trichinosis in the human body furnishes an interesting study, owing to its rarity in this country, and the cases now in the hospital are being carefully watched by medical experts. There are several kinds of trichinæ known to exist, but the particular parasite in question is usually found coiled like a snake; hence its full name of trichinæ spiralis. There are male and female of these and the young are born alive, not being hatched from eggs, as is the case with most parasites

The trichinæ being taken into the human stomach in insufficiently cooked pork, the meat is digested, leaving the parasites at liberty. They then grow rapidly until they attain a length of from one-sixteenth to one-eighth of an inch, and within three or four days begin to reproduce, each female giving birth to thousands of young. The young tricinæ attain their full growth in about seven days, after which they bore through the small intestines in which they are born and lodge themselves in the nearest muscles, whence they spread in time to all the muscles of the body. They find their way into the small muscles of the ear, impairing the hearing, or into the muscles of the eye, until the patient is compelled to hold that organ in one position to escape pain. They fill the muscles of the arms and legs until the least tension or movement causes torture, and the patient lies with legs and arms drawn up to relax the muscles and lessen the pain. So far no case is known where they have attacked the muscles of the heart, but they are frequently found in the muscular tissue of the lungs, where they cause bronchitis. The fatal termination of the disease is usually the result of bronchitis brought about in this way, or of the high fever produced by this army of parasites boring their way through the muscles of the entire body.

When they begin to propagate in the stomach or intestines irritation and inflammation follow, causing the vomiting and purging, and this continues, accompanied by intense pain in the abdomen, so long as they remain there. If taken in time they may be driven out by powerful emetics and purgatives, but this must be done within a day or two. Glycerine, or a solution of carbolic acid in glycerine, given in capsules is considered a successsul remedy at this stage of the disease. The trichinæ propagate only in the intestines. If not driven out or destroyed there they soon bore through into the muscles of the back, and it is here the first muscular pains are felt. Having once entered the muscles they are beyond the reach of medical skill, and any attempt to destroy them would be more likely to kill the patient instead. All that can be done is to relieve the patient, leaving the disease to run its course.

After six or eight weeks of this burrowing the trichina dies, and a little capsule forms about it, much like a cocoon about a caterpillar. This is at first a soft, membranous sack, but afterwards changes to a chalky cyst. Doctors sometimes find these cysts while dissecting subject showing that the persons had trichinosis and recovered.

Dr. Charles E. Hackley, of No. 63 West Thirty-sixth street, is treating the Weitzel family and carefully watching each stage of the disease. The patients were taken to the hospital last Tuesday, which was over two weeks after they had been taken sick. The trichinæ had by that time entered the muscles and were beyond the reach of medicines. Under the circumstances the doctor has confined his treatment entirely to the use of opiates and to reducing the fever. Sleeplessness is one of the most marked symptoms and opiates are therefore necessary The sleeplessness is accompanied by high fever and pain, the muscles being swollen and tender to the slightest touch. In most cases the temperature reaches 105 degrees. To reduce the fever Dr. Hackley is using a new remedy, antipyrine, a product of coal oil, which so far has proved very successful. This is given internally.

On Friday last the doctor cut from the arm of one of the patients a piece of muscular tissue about the size of a pin's head, and upon subjecting it to a microscopic examination, found a female trichinæ contained therein, thus proving that the diagnosis already given is correct. He declined positively to state which members of the family were not likely to recover, as they would be certain to hear of it and the knowledge might have a bad effect. The fever and pain, he says, will undoubtedly continue until the trichinæ become encysted or death relieves the sufferers.

